# Emerging themes and technologies in multi-organismal approaches to understanding mycorrhizal functioning

**DOI:** 10.1093/jxb/erag238

**Published:** 2026-08-12

**Authors:** David Johnson, Joanna Weremijewicz, Alan Wanke

**Affiliations:** Lancaster Environment Centre, Lancaster University, Bailrigg LA1 4YQ, UK; Department of Biology, North Central College, 30 N. Brainard St., Naperville, IL 60563, USA; Sainsbury Laboratory, 47 Bateman Street, Cambridge CB2 1LR, UK

**By sustaining plant primary production, the mycorrhizal symbiosis is crucial for regulating life on earth, and in driving numerous critical ecosystem processes and functions (Smith and Read, 2008). Increasingly, attention is turning to managing or utilizing mycorrhizal fungi to address key societal challenges such as sustainable food and timber production, woodland expansion, ecosystem restoration, and resilience against climatic extremes. Consequently, there is growing recognition of the need to consider the function and diversity of mycorrhizal symbioses from a broad perspective that includes the myriad interactions occurring across trophic levels, use of emerging technologies, and even the redefinition of our current understanding of the broad types of mycorrhiza found on Earth.**

Over the past 5 years, significant progress has been made in understanding how the mycorrhizal symbioses contribute to the challenges described above, and much of this progress has depended greatly on advances in discovery-led ‘fundamental’ science. A critical area of work tackles one of the most fundamental aspects of mycorrhizal functioning—mineral nutrient acquisition. Since the earliest work on mycorrhizas (e.g. Baylis, 1967) highlighted the key roles of fungal partners in capturing mineral nutrients (mainly plant growth-limiting nitrogen and phosphorus) from soil, hundreds of studies have emphasized nutrient capture and transfer as a central process underpinning the evolution and function of these symbioses (Smith and Read, 2008). Given the vast body of past work, it is perhaps surprising, therefore, that fundamental discoveries continue to reshape our understanding of how mycorrhizal fungi acquire nutrients. One important development is the recognition that bacteria proliferating in the mycorrhizosphere play key roles in mobilizing nutrients that are subsequently taken up by mycorrhizal hyphae. Plants rapidly allocate recently fixed carbon to their fungal partners (Johnson *et al*., 2002), and some of this carbon is exuded by hyphae and utilized as a primary energy source by hyphal-associated bacteria (Kakouridis *et al*., 2024). This energy-rich substrate facilitates microbial mineralization of complex nutrient forms, such as organic phosphorus (Wang *et al*., 2023), that can be transported to the host plant via the mycorrhizal fungus (Zhao *et al*., 2024). Thus, an emerging picture is one of a nutrient-driven symbiotic continuum spanning taxonomic kingdoms from soil bacteria through fungi to plants.

The nutritional toolkit available to plants through fungal symbioses extends beyond the well-studied types of mycorrhizal interactions. New insights into the function and diversity of Mucoromycotina ‘fine root endophytes’ (MFREs) have revealed that these fungi offer host plants nutritional benefits comparable with those provided by arbuscular mycorrhizal fungi. Historically, observations of ‘fine root endophytes’ in roots were often dismissed compared with arbuscular mycorrhizal fungi, and subsequently the fungi were referred to as *Glomus tenue*—placing them with arbuscular mycorrhizal fungi within the Glomeromycotina clade. We now know that MFREs are phylogenetically and functionally distinct: they belong to the Endogonales (Sinanaj *et al*., 2021) and can provide host plants, spanning liverworts to vascular plants, with both nitrogen and phosphorus (Hoysted *et al*., 2023). Moreover, MFREs have the capacity to utilize organic forms of nitrogen, such as glycine, mineralizing the amino acid to access its carbon while transferring nitrogen to the host. In return, plants allocate recent photosynthate to the fungi (Howard *et al*., 2024). An intriguing aspect of MFREs is that they often co-exist alongside arbuscular mycorrhizal fungi within the same host, raising questions about the potential complementarity between the two fungal guilds, their competition for host-derived carbon, and their relative susceptibilities to perturbations such as drought or soil nutrient enrichment.

Mycorrhizal fungi produce extra-radical mycelium that is highly efficient at exploring the heterogeneous environments characteristic of most soils. These networks of hyphae can be considered at different scales. At their simplest, they can be described in terms of the hyphal density of an individual fungus, or as interaction networks between individual fungi and individual plants within or among populations and communities (Ajaz *et al*., 2026). Another compelling way to consider these fungal networks is through the manner in which they influence neighbouring plants, either directly by forming a continuous, physically connected hyphal network, or indirectly by influencing the properties of soils in the mycorrhizosphere or rhizosphere of neighbours (Rillig *et al.,* 2025). These so-called ‘common mycorrhizal networks’ (CMNs) with or without hyphal continuity generate many fascinating outcomes, including effects on plant community dynamics (Workman and Cruzan, 2016), animal behaviour (Babikova *et al*., 2013), carbon and mineral nutrient distribution (Weremijewicz *et al*., 2016), and potentially ecosystem resilience (Alaux *et al*., 2021). A key challenge in advancing our understanding of fungal network function is visualizing hyphae and hyphal continuity in soil. New extraction and imaging technologies are needed to bridge the persistent gap between functional and taxonomic characterization of mycorrhizas—a gap that remains central to our field. In this Special Issue, we bring together papers falling under the broad heading of ‘emerging themes and technologies in multi-organismal approaches to understand mycorrhizal functioning’. The collection grew out of lively and inspiring discussions and presentations at the 12th International Conference on Mycorrhiza (ICOM12) in Manchester, UK, in 2024.

## Conceptual advances in mycorrhizal symbioses

The recent recognition that MFREs are widespread and form nutritional symbioses with many herbaceous vascular plants and non-vascular plants requires a robust conceptual and taxonomic framework for these fungi. The Viewpoint by Howard *et al*. (2026) illustrates how terminological inconsistency in MFRE nomenclature compounds over time and becomes increasingly costly to resolve. The authors argue that adopting the name MFREs, as opposed to competing terms, makes visible how names, arising from scientific uncertainty at different historical moments, can fragment the literature, impede meta-analyses, and create barriers to entry for new researchers. Labelling MFREs as a form of arbuscular mycorrhizal fungi based on superficial morphological or functional similarities risks importing assumptions (about colonization mechanisms, nutrient exchange interfaces, and signalling pathways) that may not hold and that could misdirect experimental design. Maintaining clear terminological boundaries between phylogenetically distinct groups helps ensure that apparent similarities are investigated rather than assumed.

The recognition of the individuality of mycorrhizal plant and fungal partners is also conceptually important. Historically, many researchers have taken a ‘phytocentric’ view of the mycorrhizal symbiosis, but increasingly this perspective has been challenged to consider a mycocentric perspective that emphasizes the fungi as independent partners in the symbiosis (Robinson and Fitter, 1998; Babikova *et al*., 2013). Veresoglou (2026) brings together these phytocentric and mycocentric views and considers them from an evolutionary perspective. For example, he argues that an explicit ‘holobiont’ view, whereby both partners are considered together, may not be appropriate, and puts forward a hierarchy of hypotheses that may be more suitable to test using mycocentric, phytocentric, and holobiont perspectives. Such integrative thinking connects to the question of how mycorrhizal partners communicate. Lee *et al*. (2026) review emerging evidence that common mycorrhizal networks serve as conduits for inter-plant defence signalling, transmitting stress-related cues—including phytohormones, reactive oxygen species, and small RNAs transported via extracellular vesicles—from attacked donor plants to neighbouring receivers. Critically, the molecular identity of the signals traversing common mycorrhizal networks remains largely unknown, and whether they represent direct plant-to-plant transfer or a ‘two-step translation’ involving fungal intermediaries is an open question with important implications for how we conceptualize communication within mycorrhizal systems.

## Visualizing and measuring mycorrhizal fungi

There is no doubt that the challenges of working in soil have limited our ability to understand the function and diversity of mycorrhizal fungi. However, new technologies and our ability to integrate them promise to help overcome these challenges. While extracting hyphae from soil remains fundamental, current methods are labour-intensive and can compromise associated microbial communities. Metzen and Bucher (2026) develop a semi-automated sieving and sucrose centrifugation approach to extract and enumerate hyphae, while maintaining the viability of microbes associated with the fungi. Their approach is 2- to 2.5-fold faster than previous methods and more efficient at extracting hyphae from natural substrates, while also reducing operator-dependent variability. Critically, by reducing the loss of diversity of microbes associated with hyphae, the method advances our ability to study mycorrhizal–microbial interactions.

Complementing such extraction-based approaches, Birt *et al*. (2026) review how advanced imaging approaches, such as micro-computed tomography, developed primarily for medical research, offer scope to integrate functional analyses (such as visualizing the movement of mineral nutrients or other larger functionally important molecules such as fatty acids) with fungal and plant root network architecture. The ability to visualize function and morphology non-invasively has obvious advantages over more destructive techniques and permits more effective testing of how different soil properties may impact mycorrhizal function. An area where these visualization approaches could be further developed is in the identification of molecules involved in plant-to-plant signalling via fungal networks (Babikova *et al*., 2013), which would help to more effectively harness these networks in agricultural systems (Lee *et al*., 2026).

Capturing the functionality of mycorrhizal fungi, especially arbuscular mycorrhizal fungi, at the scale of single cells is fraught with technical difficulties. Ferreras-Garrucho *et al*. (2026) help resolve some of these challenges and review methodological pipelines for dual plant–fungal transcriptomics. They provide a unified framework showing how single-cell and single-nucleus sequencing, spatial transcriptomics, cell-state-specific, tagging and live-cell biosensors can be combined to enable the interrogation of both plant and fungal gene expression simultaneously. Moreover, single-cell resolution is vital to understand how plants interact with their biotic partners at spatial scales at which these interactions occur.

## Resource capture

The mycorrhizal symbiosis provides host plants with critical growth-limiting nutrients, primarily nitrogen and phosphorus. Access to nitrogen is particularly important in boreal forests, where low temperatures and acidic pH conditions limit microbial mineralization, and instead complex organic forms dominate the total soil nitrogen pool. Emerging evidence suggests that ectomycorrhizal fungi, the dominant mycorrhizal type in boreal forests, can enforce plant nitrogen limitation in these systems (Franklin *et al*., 2014). The addition of nitrogen from fertilization or atmospheric deposition could therefore profoundly alter the function of the mycorrhizal symbioses in these systems. This topic is reviewed by Cox (2026), who synthesizes our current understanding of how even modest inputs of nitrogen can reshape ectomycorrhizal fungal communities and alter how plants allocate carbon below-ground to their fungal partners, with consequences for litter decomposition, biogeochemical cycling, and carbon sequestration.

Nitrogen limitation, however, is not restricted to ectomycorrhizal systems. While arbuscular mycorrhiza are traditionally considered to be specialists in phosphorus-limited systems, this view belies the breadth of ecosystems in which this mycorrhizal type prevails. Zhang *et al*. (2026) highlight the varying impacts different arbuscular mycorrhizal fungal species can have on plant performance under nitrogen limitation. Their work provides striking evidence of contrasting effects of arbuscular mycorrhizal species identity and emphasizes the gradient of benefits offered by mycorrhizal fungi, which was first conceptualized nearly 30 years ago (Johnson *et al*., 1997).

It is likely that some of the variation in plant benefits from mycorrhizal fungi occurs indirectly through interaction with hyphosphere bacteria. Sun *et al*. (2026) demonstrate that a hyphosphere keystone bacterial taxon, *Massilia*, adopts host-dependent functional strategies that promote the growth of arbuscular mycorrhiza and mineralization of organic phosphorus through phosphatase production. This and other recent discoveries (e.g. Wang *et al*., 2023) offer opportunities to enhance crop production, as well as to test some of the hypotheses highlighted by Veresoglou (2026) from a holobiont perspective.

## Conclusion

This Special Issue highlights some of the key technological, conceptual, and fundamental advances that will help improve our understanding of mycorrhizal functioning across scales and ecological contexts. These advances are critical to tackling some of society’s most pressing challenges, including increasing sustainable food production, sequestering carbon to mitigate the effects of global climate change, and protecting terrestrial biodiversity. The body of work summarized here emphasizes the importance of multifaceted approaches to understanding the mycorrhizal symbiosis, both as a requirement to understand natural processes spanning trophic levels and to inspire new approaches for studying mycorrhizal functioning.
